# Special Issue: Coinage Metal (Copper, Silver, and Gold) Catalysis

**DOI:** 10.3390/molecules21060746

**Published:** 2016-06-08

**Authors:** Sónia Alexandra Correia Carabineiro

**Affiliations:** Laboratório de Catálise e Materiais (LCM), Laboratório Associado LSRE-LCM, Departamento de Engenharia Química, Faculdade de Engenharia, Universidade do Porto, Rua Dr Roberto Frias s/n, 4200-465 Porto, Portugal; scarabin@fe.up.pt; Tel.: +351-22-0414907

**Keywords:** copper, silver, gold, catalysis, selectivity

## Abstract

The subject of catalysis by coinage metals (copper, silver, and gold) comes up increasingly day-by-day. This Special Issue aims to cover the numerous aspects of the use of these metals as catalysts for several reactions. It deals with synthesis and characterization of copper, silver and gold based catalysis, their characterization and use, both for heterogeneous and homogeneous catalysis, and some of their potential applications.

Catalysts are materials that allow important reactions to be more selective, faster and require less energy. Coinage metals (copper, silver, and gold) are often found as important components of most catalysts and are known for their high activity and important properties. Thus the demand for research on this topic is continuously increasing.

This Special Issue aims to cover several aspects of the usage of Cu, Ag and Au as catalysts for both homogenous and heterogeneous catalysis. It consists of nine important “cutting-edge” open access papers available to all online.

The role of sub-nanometric Ag species (on Fe, Mg and Ce modified TiO_2_ materials) on catalytic CO oxidation is studied by several techniques in [1]. The observed phenomena cannot be explained only considering the 1–13 nm Ag particle size distributions measured by HRTEM; the existence of Ag species with size < 1 nm, non-visible in HRTEM, and their interaction with the supports, needs also to be taken into account.

Silver and copper are reported as catalysts, in a new and efficient way, for the cycloaddition of propargyl-substituted dihydroisoindolin-1-one with arylnitrile oxides to produce novel 3,5-disubstituted isoxazoles of the ethyl-2-benzyl-3-oxo-1-((3-arylisoxazol-5yl)methyl)-2,3-dihydro-1*H*-isoindole-1-carboxylate type [2]. Yields are significantly improved with the presence of Cu^I^ as catalyst compared to the uncatalyzed reaction, without changing the selectivity.

Aroylhydrazone Cu(II) complexes in keto form are reported as catalysts for the peroxidative oxidation of cyclohexane to cyclohexyl hydroperoxide, cyclohexanol and cyclohexanone, under mild conditions [3]. The activity exhibited by those complexes in the absence of any additive, is higher than that shown by other efficient Cu^II^ systems reported in the literature. 

Copper is also used as a catalyst, deposited on different lanthanide-doped cerium oxides (Ce_0.5_Ln_0.5_O_1.75_, where Ln: Gd, La, Pr, Nd) for the oxidation of ethyl acetate, a harmful volatile organic compound [4]. The best catalytic performance was obtained for Cu/CeO_2_ and Cu/Ce_0.5_Pr_0.5_O_1.75_ samples. A strong correlation was found between the catalytic performance and the redox properties of the samples, in terms of reducibility and lattice oxygen availability. 

Gold incorporated on a mesoporous silica thin film is reported as a robust surface enhanced Raman scattering (SERS) and catalytically active substrate for the reduction of *p*-nitrophenol to *p*-aminophenol with sodium borohydride [5]. The reported approach can be used for making catalytically active metal nanoparticle-silica that can potentially bridge the material gap between real catalyst systems and surface science studies.

A gold-modified silicalite is reported as catalyst for the conversion of ethanol into acetaldehyde or acetic acid [6]. This material might offer a promising eco-friendly way for the production of these two important products and has the potential to become a sustainable alternative to presently used methods. The unloaded silicalite-1 also catalyzes the dehydration of ethanol to diethylether or ethane.

The effect of (Ce, La and Fe oxide) modifiers and redox treatments (at different temperatures) on the electronic states of 1 nm gold nanoparticles supported on silica is also studied by means of Fourier transformed infrared (FTIR) spectra of adsorbed CO at room temperature [7]. It is shown that the electronic state of gold in small (~1 nm) particles is sensitive to the nature of the support surface, as well as to temperature and atmosphere of redox pretreatments. However, Au^0^ remains stable, independent of additives and redox pretreatments, showing no significant effect of such treatments on the electronic properties of larger nanoparticles (3–7 nm).

The causes for the deactivation and reactivation of Au/TiO_2_ catalysts after long-term storage and redox treatments are also reported [8]. It is stated that the main reason for the deactivation and reactivation is the variation in the electronic state of the supported gold, namely, the amount of Au^+^ ions. Thus the most active samples are those with the highest proportion of singly charged gold ions, while materials with a high content of Au^+3^ are inactive at low-temperatures. Higher stabilization can be achieved through modification of TiO_2_ with transition metals oxides (namely La_2_O_3_). 

Finally, the electrochemical characterization (by cyclovoltammetry CV and electrochemical impedance spectroscopy EIS) of Au–Pd systems is reported to determine the presence of an electronic interaction between the two metals [9]. The higher activity of the AuPd bimetallic system in the liquid phase oxidation of glycerol is due to a compromise between the stability of the oxidic species (decreased with respect to Pd) and the facility of hydride formation (increased with respect to Au). This balance creates the observed synergistic effect between Au and Pd.

I thank all the authors who contributed to this Special Issue and the staff members of MDPI for their editorial support. I trust readers will find the papers herein published helpful and interesting examples of the use of coinage metals in catalysis.

## References

[1] Kotolevich Y., Kolobova E., Khramov E., Cabrera Ortega J., Farías M., Zubavichus Y., Zanella R., Mota-Morales J., Pestryakov A., Bogdanchikova N. (2016). Identification of Subnanometric Ag Species, Their Interaction with Supports and Role in Catalytic CO Oxidation. Molecules.

[2] Rammah M., Gati W., Mtiraoui H., Rammah M., Ciamala K., Knorr M., Rousselin Y., Kubicki M. (2016). Synthesis of Isoxazole and 1,2,3-Triazole Isoindole Derivatives via Silver- and Copper-Catalyzed 1,3-Dipolar Cycloaddition Reaction. Molecules.

[3] Sutradhar M., Alegria E., Guedes da Silva M., Martins L., Pombeiro A. (2016). Aroylhydrazone Cu(II) Complexes in keto Form: Structural Characterization and Catalytic Activity towards Cyclohexane Oxidation. Molecules.

[4] Carabineiro S., Konsolakis M., Marnellos G., Asad M., Soares O., Tavares P., Pereira M., Órfão J., Figueiredo J. (2016). Ethyl Acetate Abatement on Copper Catalysts Supported on Ceria Doped with Rare Earth Oxides. Molecules.

[5] Sunil Sekhar A., Vinod C. (2016). Gold Incorporated Mesoporous Silica Thin Film Model Surface as a Robust SERS and Catalytically Active Substrate. Molecules.

[6] Falletta E., Rossi M., Teles J., Della Pina C. (2016). Clean Transformation of Ethanol to Useful Chemicals. The Behavior of a Gold-Modified Silicalite Catalyst. Molecules.

[7] Martynyuk O., Kotolevich Y., Vélez R., Cabrera Ortega J., Tiznado H., Zepeda Partida T., Mota-Morales J., Pestryakov A., Bogdanchikova N. (2016). On the High Sensitivity of the Electronic States of 1 nm Gold Particles to Pretreatments and Modifiers. Molecules.

[8] Kolobova E., Kotolevich Y., Pakrieva E., Mamontov G., Farías M., Bogdanchikova N., Cortés Corberán V., Pestryakov A. (2016). Causes of Activation and Deactivation of Modified Nanogold Catalysts during Prolonged Storage and Redox Treatments. Molecules.

[9] Pifferi V., Chan-Thaw C., Campisi S., Testolin A., Villa A., Falciola L., Prati L. (2016). Au-Based Catalysts: Electrochemical Characterization for Structural Insights. Molecules.

